# The landscape of human STR variation

**DOI:** 10.1101/gr.177774.114

**Published:** 2014-11

**Authors:** Thomas Willems, Melissa Gymrek, Gareth Highnam, The 1000 Genomes Project Consortium, David Mittelman, Yaniv Erlich

**Affiliations:** 1 Whitehead Institute for Biomedical Research, Cambridge, Massachusetts 02142, USA;; 2 Computational and Systems Biology Program, MIT, Cambridge, Massachusetts 02139, USA;; 3 Harvard-MIT Division of Health Sciences and Technology, MIT, Cambridge, Massachusetts 02139, USA;; 4 Program in Medical and Population Genetics, Broad Institute of MIT and Harvard, Cambridge, Massachusetts 02142, USA;; 5 Department of Molecular Biology and Diabetes Unit, Massachusetts General Hospital, Boston, Massachusetts 02114, USA;; 6 Virginia Bioinformatics Institute and Department of Biological Sciences, Virginia Tech, Blacksburg, Virginia 24061, USA;; 7 Gene by Gene, Ltd., Houston, Texas 77008, USA

## Abstract

Short tandem repeats are among the most polymorphic loci in the human genome. These loci play a role in the etiology of a range of genetic diseases and have been frequently utilized in forensics, population genetics, and genetic genealogy. Despite this plethora of applications, little is known about the variation of most STRs in the human population. Here, we report the largest-scale analysis of human STR variation to date. We collected information for nearly 700,000 STR loci across more than 1000 individuals in Phase 1 of the 1000 Genomes Project. Extensive quality controls show that reliable allelic spectra can be obtained for close to 90% of the STR loci in the genome. We utilize this call set to analyze determinants of STR variation, assess the human reference genome’s representation of STR alleles, find STR loci with common loss-of-function alleles, and obtain initial estimates of the linkage disequilibrium between STRs and common SNPs. Overall, these analyses further elucidate the scale of genetic variation beyond classical point mutations.

Short tandem repeats (STRs) are abundant repetitive elements comprised of recurring DNA motifs of 2–6 bases. These loci are highly prone to mutations due to their susceptibility to slippage events during DNA replication (Ellegren 2004). To date, STR mutations have been linked to at least 40 monogenic disorders (Pearson et al. 2005; Mirkin 2007), including a range of neurological conditions such as Huntington’s disease, amyotrophic lateral sclerosis, and certain types of ataxia. Some disorders, such as Huntington’s disease, are triggered by the expansion of a large number of repeat units. In other cases, such as oculopharyngeal muscular dystrophy, a pathogenic allele is only two repeat units from the wild-type allele (Brais et al. 1998; Amiel et al. 2004). In addition to Mendelian conditions, multiple studies have suggested that STR variations contribute to an array of complex traits (Gemayel et al. 2010), ranging from the period of the circadian clock in *Drosophila* (Sawyer et al. 1997) to gene expression in yeast (Vinces et al. 2009) and splicing in humans (Hefferon et al. 2004; Sathasivam et al. 2013).

Beyond their importance to medical genetics, STR variations convey high information content due to their rapid mutations and multiallelic spectra. Population genetics studies have utilized STRs in a wide range of methods to find signatures of selection and to elucidate mutation patterns in nearby SNPs (Tishkoff et al. 2001; Sun et al. 2012). In DNA forensics, STRs play a significant role as both the United States and the European forensic DNA databases rely solely on these loci to create genetic fingerprints (Kayser and de Knijff 2011). Finally, the vibrant genetic genealogy community extensively uses these loci to develop impressive databases containing lineages for hundreds of thousands of individuals (Khan and Mittelman 2013).

Despite the utility of STRs, systematic data about their variation in the human population is far from comprehensive. Currently, most of the genetic information concerns a few thousand loci that were part of STR linkage and association panels in the pre-SNP-array era (Broman et al. 1998; Tamiya et al. 2005) and several hundred loci involved in forensic analysis, genetic genealogy, or genetic diseases (Ruitberg et al. 2001; Pearson et al. 2005). In total, there are only 5500 loci under the microsatellite category in dbSNP139. For the vast majority of STR loci, little is known about their normal allelic ranges, frequency spectra, and population differences. This knowledge gap largely stems from the absence of high-throughput genotyping techniques for these loci (Jorgenson and Witte 2007). Capillary electrophoresis offers the most reliable method to probe these loci, but this technology scales poorly. More recently, several studies have begun to genotype STR loci with whole-genome sequencing data sets obtained from long read platforms such as Sanger sequencing (Payseur et al. 2011) and 454 Life Sciences (Roche) (Molla et al. 2009; Duitama et al. 2014). However, due to the relatively low throughput of these platforms, these studies analyzed STR variations in only a few genomes.

Illumina sequencing has the potential to profile STR variations on a population-scale. However, STR variations present significant challenges for standard sequence analysis frameworks (Treangen and Salzberg 2012). In order to reduce computation time, most alignment algorithms use heuristics that reduce their tolerance to large indels, hampering alignment of STRs with large contractions or expansions. In addition, due to the repetitive nature of STRs, the PCR steps involved in sample preparation induce in vitro slippage events (Hauge and Litt 1993). These events, called stutter noise, generate erroneous reads that mask the true genotypes. Because of these issues, previous large-scale efforts to catalog genetic variation have omitted STRs from their analyses (The 1000 Genomes Project Consortium 2012; Tennessen et al. 2012; Montgomery et al. 2013), and early attempts to analyze STRs using the 1000 Genomes Project data mainly focused on exonic regions (McIver et al. 2013) or extremely short STR regions in a relatively small number of individuals based on the native indel call set (Ananda et al. 2013).

In our previous studies, we created publicly available programs that specialize in STR profiling using Illumina whole-genome sequencing data (Gymrek et al. 2012; Highnam et al. 2013). Recently, we employed one of these tools (lobSTR) to accurately genotype STRs on the Y chromosome of anonymous individuals in the 1000 Genomes Project to infer their surnames (Gymrek et al. 2013), demonstrating the potential utility of STR analysis from Illumina sequencing. Here, we used these tools to conduct a genome-wide analysis of STR variation in the human population using sequencing data from Phase 1 of the 1000 Genomes Project.

## Results

### Identifying STR loci in the human genome

The first task in creating a catalog of STR variation is to determine the loci in the human reference that should be considered as STRs. This problem primarily stems from the lack of consensus in the literature as to how many copies of a repeat distinguish an STR from genomic background (Leclercq et al. 2007; Fondon et al. 2012; Schaper et al. 2012). For example, is (AC)_2_ an STR? What about (AC)_3_ or (AC)_10_? Furthermore, as sporadic bases can interrupt repetitive DNA sequences, purity must also be taken into account when deciding whether a locus is a true STR.

We used a quantitative approach to identify STR loci in the reference genome. Multiple lines of study have proposed that the birth of an STR is a relatively rare event with complex biology (Ellegren 2004; Buschiazzo and Gemmell 2006; Oliveira et al. 2006; Gemayel et al. 2010; Kelkar et al. 2011; Ananda et al. 2013). The transition from a proto-STR to a mature STR requires nontrivial mutations, such as the arrival of a transposable element, slippage-induced expansion of the proto-STR, or precise point mutations that destroy nonrepetitive gaps between two short repeat stretches. Based on these observations, it was suggested that randomly shuffled DNA sequences should rarely produce mature STR sequences and can therefore be used as negative controls for STR discovery algorithms (Gemayel et al. 2010; Schaper et al. 2012). We utilized this approach to identify STR loci in the human genome while controlling the false positive rate (Supplemental Fig. 1; Supplemental Methods). We first integrated the purity, composition, and length of putative STRs in the genome into a score using Tandem Repeats Finder (TRF) (Benson 1999). Then, we generated random DNA sequences using a second-order Markov chain with similar properties to the human genome (i.e., nucleotide composition and transition frequencies). We tuned the TRF score threshold such that only 1% of the identified STR loci in our collection were expected to be false positives. The resulting score thresholds were in good qualitative agreement with those previously produced using a variety of alternative experimental and analytical methods (Supplemental Methods; Lai and Sun 2003; Kelkar et al. 2010; Fondon et al. 2012). We then evaluated the false negative rate of our catalog using two methods (Supplemental Methods). First, we collected a preliminary call set of repeat number variability across the human population with a highly permissive definition of STR loci. We found that our catalog misses only ∼1% of loci that exhibited repeat variability in this call set (Supplemental Table 1). Second, we collated a set of about 850 annotated bona fide STR loci, mainly from the CODIS forensic panel and Marshfield linkage panel. Only 12 (1.4%) of these markers were not included in the catalog based on the TRF score threshold. The results of the two validation methods suggest that our catalog includes ∼99% of the true STRs in the genome and has a false negative rate of ∼1%.

Overall, our STR reference includes ∼700,000 loci in the human genome. About 75% of these loci are di- and tetra-nucleotide STRs, whereas the remaining loci are tri-, penta-, and hexa-nucleotide STRs (Supplemental Table 2). Approximately 4500 loci overlap coding regions, 80% of which have either trimeric or hexameric repeat units. In addition, our reference contains a roughly equal proportion of interrupted and uninterrupted microsatellites.

### Profiling STRs in the 1000 Genomes Project samples

We collected variations for these 700,000 STR loci using 1009 individuals from Phase 1 of the 1000 Genomes Project (Methods; Supplemental Table 3). These samples span populations from five continents and were subject to low coverage (∼5×) whole-genome sequencing using 76-bp and 100-bp Illumina paired-end reads. In addition, high coverage exome sequencing data were available for 975 of these samples and were integrated with the whole-genome raw sequencing files.

We tested two distinct STR genotyping pipelines designed to analyze high-throughput sequencing data, namely lobSTR (Gymrek et al. 2012) and RepeatSeq (Highnam et al. 2013). Briefly, lobSTR utilizes the nonrepetitive regions surrounding STRs to align reads and assess their allele lengths, whereas RepeatSeq utilizes Bayesian model selection to genotype previously aligned STR-containing reads. Despite significant methodological differences, the STR genotypes from the two tools were quite concordant and matched for 133,375,900 (93%) of the 143,428,544 calls that were reported by both tools. We tested multiple methods to unify the two call sets in order to further improve the quality (Supplemental Fig. 2; Supplemental Methods). However, none of these integration methods improved the accuracy. Since the lobSTR calls showed better quality for highly polymorphic STRs, we proceeded to analyze STR variations using only this call set.

**Figure 1.**
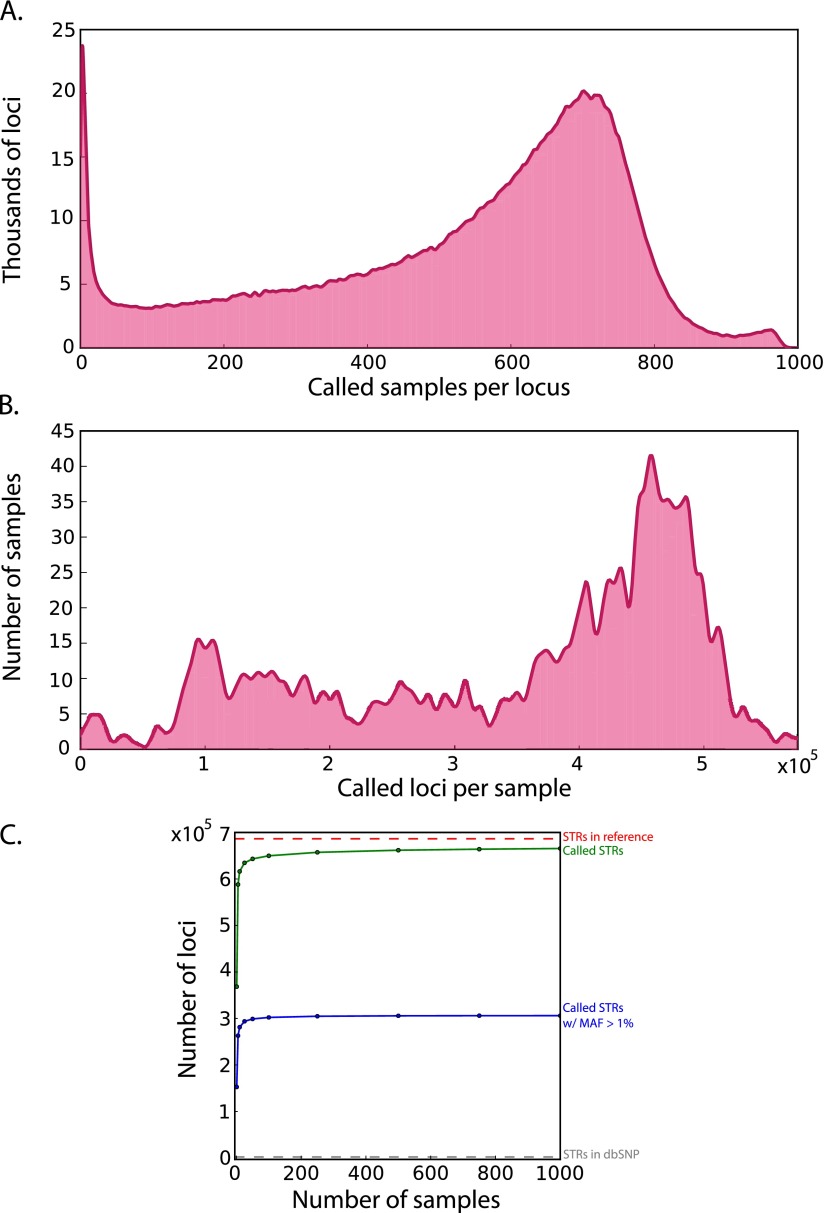
Call set statistics. (*A*) Distribution of the number of called samples per locus. The average is 528 samples per STR with a standard deviation of 231. (*B*) Distribution of the number of called loci per sample. The average is 349,892 STRs per sample with a standard deviation of 145,135. (*C*) Saturation curves for the catalog. The number of called loci (green) rapidly approaches the total number of STRs in the genome (red line). The number of called loci with a MAF > 1% (blue) saturates after 100 samples and far exceeds the number of STR variants in dbSNP (gray line close to the *x*-axis).

On average, we collected STR genotypes for ∼530 individuals per locus (Fig. 1A) and 350,000 STR loci per individual (Fig. 1B), accumulating a total of about 350 million STR genotypes in the catalog. We examined the marginal increase in the number of covered STR loci as a function of sample size (Methods; Fig. 1C). Our analysis shows that after analyzing 100 samples, there is a negligible increase in the number of genotyped STRs. However, even with all of the data, 3% of STR loci are persistently absent from the catalog. The average reference allele length of the missing STR loci was 182 bp compared to 31 bp for the rest of the reference, suggesting that the missing STR loci have allele lengths beyond the read length of Illumina sequencing. We also examined the marginal increase of polymorphic STR loci with minor allele frequencies (MAF) > 1%. Again, we observed an asymptote after ∼100 samples. These saturation analyses suggest that with the current sample size, the STR variation catalog virtually exhausted all loci with MAF > 1% that can be observed with 100-bp Illumina reads and our analysis pipeline.

The full catalog of STR variations is publicly available at http://strcat.teamerlich.org in VCF format. In addition, the website provides a series of graphical interfaces to search for STR loci with specific biological properties, obtain summary statistics such as allelic spectra and heterozygosity rates, and view the supporting raw sequencing reads.

### Quality assessment

**Figure 2.**
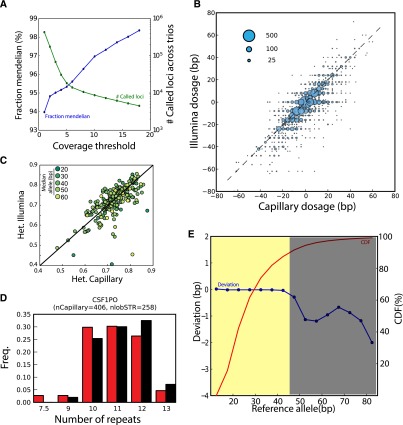
Quality assessments of the STR catalog. (*A*) Consistency of lobSTR calls with Mendelian inheritance. The blue line denotes the fraction of STR loci that followed Mendelian inheritance as a function of the read coverage threshold. The green line denotes the total number of calls in the three trios that passed the coverage threshold. (*B*) Concordance between lobSTR and capillary electrophoresis genotypes. The STR calls were taken from the highly polymorphic Marshfield panel. The dosage is reported as the sum of base pair differences from the hg19 reference. The area of each bubble is proportional to the number of calls of the dosage combination, and the broken line indicates the diagonal. (*C*) Comparison of heterozygosity rates for Marshfield panel STRs. The color denotes the length of the median allele of the STR (dark-short; light-long). (*D*) A comparison of allelic spectra obtained by lobSTR and capillary electrophoresis for a CODIS marker in European individuals. (Red) lobSTR; (black) capillary electrophoresis. nlobSTR and nCapillary indicate the number of alleles called in the respective call sets. (*E*) The reliable range of lobSTR allelic spectra. The figure presents the median deviation of the lobSTR calls from hg19 as function of the reference allele length (blue curve). Negative deviations indicate a potential preference toward ascertaining shorter alleles. STRs with reference alleles of up to ∼45 bp show very minimal deviations (yellow region) and are expected to display unbiased frequency spectra with the current read lengths. These STR loci comprise close to 90% of the total genotyped STRs in our catalog (red curve).

To initially assess the accuracy of our STR calls, we first examined patterns of Mendelian inheritance (MI) of STR alleles for three low-coverage trios present in the sample set. In total, we accumulated half a million calls. Without any read depth threshold, 94% of the STR loci followed MI (Fig. 2A). The MI rates increased monotonically with read depth, and restricting the analysis to loci with at least 10 reads increased the Mendelian inheritance to >97%.

Next, we compared the concordance between our calls and those obtained using capillary electrophoresis, the gold standard for STR calling (Methods). We focused on data sets containing Marshfield and PowerPlex Y chromosome panel genotypes that are available for a subset of the 1000 Genomes Project individuals. These panels ascertain some of the most polymorphic STR loci, testing our pipeline in a challenging scenario. The Marshfield capillary panel (Rosenberg et al. 2005) reported 5164 genotypes that overlapped with the lobSTR calls and pertained to 157 autosomal STRs and 140 individuals, whereas the PowerPlex capillary panel reported 784 genotypes that overlapped with the lobSTR calls and pertained to 17 Y-STRs and 228 individuals.

One key question is finding an adequate cost function to assess the concordance between the STR calls. In SNPs, the proportion of mismatches is a natural measure of concordance due to their binary nature. However, for STRs, this approach assigns the same penalty for missing one repeat unit and 10 repeat units. As an alternative, we focused on measuring the goodness-of-fit (*R*^2^) between the STR dosages. The dosage of an STR was defined as the sum of the number of base pairs after subtracting the reference allele. For example, if the genotype was 16 bp/18 bp and the reference allele was 14 bp, the dosage of the locus was set to 2 + 4 = 6; for hemizygous loci, the dosage was the difference from the reference allele. We focused on assessing dosage concordance because of the growing body of studies suggesting that the phenotypic impact of STRs is strongly correlated with length (Gebhardt et al. 1999; Shimajiri et al. 1999; Contente et al. 2002; Hefferon et al. 2004). *R*^2^ confers the property that the cost is proportional to the (squared) magnitude of the error in terms of length. In addition, the *R*^2^ of the dosages measures the amount of genetic variance that was recovered by lobSTR under strict additivity, which might be important for downstream association studies.

After regressing the lobSTR dosages with the capillary dosages, the resulting goodness-of-fit estimators (*R*^2^) were 0.71 for the autosomal genotypes and 0.94 for the Y chromosome genotypes (Fig. 2B; Supplemental Fig. 3). By further stratifying the autosomal calls by the capillary genotype, we found that lobSTR correctly reported 89.5% of all homozygous loci and recovered one or more alleles for 91.5% of all heterozygous loci, but only correctly reported both alleles for 12.8% of all heterozygous loci (Supplemental Table 4). For the Y chromosome, 95% of the lobSTR genotypes exactly matched the capillary genotypes for the PowerPlex Y panel (Supplemental Table 5).

Collectively, these results suggest that the individual allele lengths are relatively accurate, and that the primary source of noise is the recovery of only one STR allele for heterozygous loci, an issue known as allelic dropout. This statement is supported by the relatively good accuracy achieved for the homozygous autosomal loci and hemizygous Y chromosome loci, and the monotonically increasing relationship between heterozygote accuracy and read depth, with a heterozygote accuracy of nearly 80% achieved for loci covered by six or more reads (Supplemental Fig. 4). In general, allelic dropouts are quite expected given the relatively low sequencing coverage but are also known to be an issue in genotyping STRs with capillary electrophoresis (Pompanon et al. 2005).

We performed various analyses that demonstrate that allelic dropouts do not hamper the ability to deduce population-scale patterns of human STR variation. First, we examined the concordance of heterozygosity rates obtained from the lobSTR and the capillary calls for Marshfield STRs in three European subpopulations (CEU, GBR, and FIN). The heterozygosity rate is based on the frequency spectrum of a locus (Methods) and should be unaffected by random allelic dropout. As expected, we found that the heterozygosity rates were highly similar between the capillary and the lobSTR results (Fig. 2C). The regression slope was 0.996 and the root mean squared error (RMSE) was 0.044 based on more than 200 STRs. This analysis shows that the heterozygosity estimates obtained from our call set are relatively unbiased.

We also benchmarked the quality of population-scale patterns by comparing the allelic spectra for the Marshfield loci (Supplemental Fig. 5). We found that in most cases, the lobSTR and capillary spectra matched in the median and interdecile range of the reported allelic lengths. We also inspected the frequency spectra of STRs that are part of the forensic CODIS test panel using a similar procedure (Fig. 2D; Supplemental Fig. 6). A previous study reported the spectra of these loci by genotyping ∼200 Caucasians in the United States using capillary electrophoresis (Budowle et al. 1999). Again, these comparisons resulted in similar patterns for eight of the 10 analyzed markers. We found marked biases only for FGA and D18S51, with lobSTR reporting systematically shorter alleles. As the maximal allele sizes of these two loci are >80 bp, the long alleles are seldom spanned by the mixture of 76-bp and 100-bp Illumina reads in Phase 1, creating a bias toward shorter alleles.

We sought to further characterize potential biases toward ascertaining shorter alleles with lobSTR and the 76-bp/100-bp Illumina reads. To that end, we inspected the concordance between the lobSTR calls and the hg19 reference (Fig. 2E). The reference genome was generated by long Sanger reads and should therefore be an unbiased estimator of the most common allele in the population. In the absence of any systematic bias toward shorter alleles, the expected deviation of a lobSTR allele from the NCBI reference should be zero. On the other hand, in the presence of such a bias, the lobSTR calls should be systematically smaller than the NCBI reference and generate a negative deviation. We found that the median deviation of lobSTR was around zero for STRs with reference alleles up to 45 bp. Above this threshold, we started to observe systematic deviations toward shorter alleles. The deviation did not monotonically decrease but exhibited a local maximum around 65 bp, which presumably stems from the heterogeneity of the sequencing read lengths and the exhaustion of alleles that can be spanned by 76-bp reads. Importantly, only 10% of all loci in our catalog have a reference allele >45 bp. This implies that for the vast majority of the loci, the allelic spectra are expected to be unbiased.

### Validation using population genetics trends

**Figure 3.**
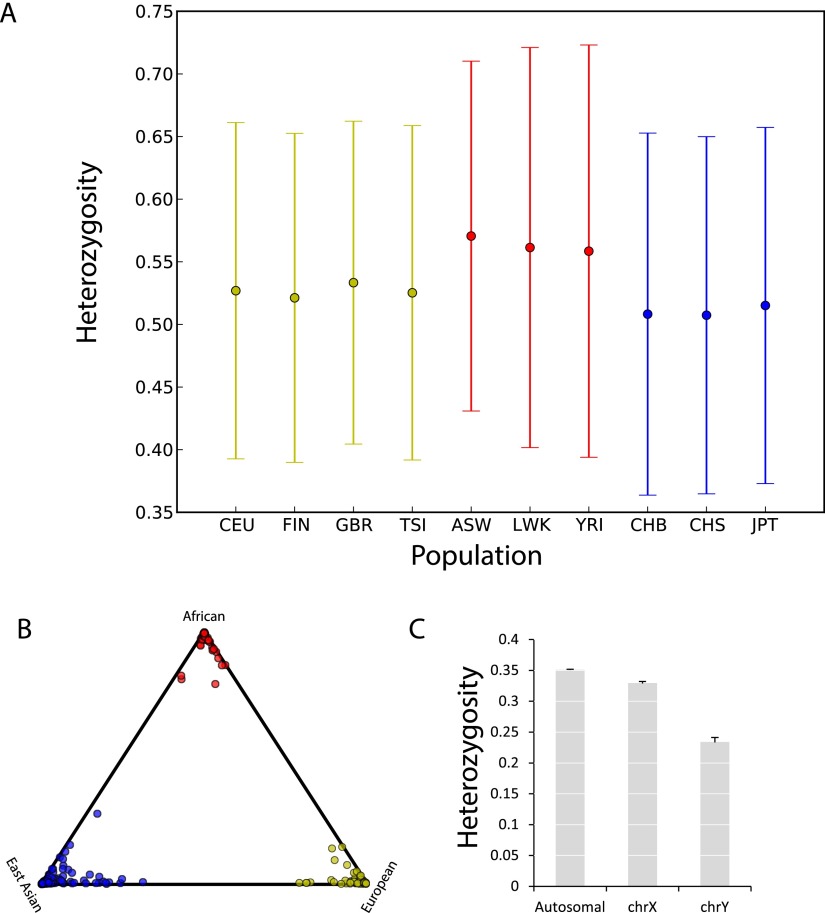
Evaluation of the STR catalog for population genetics. (*A*) Genetic diversity of the 10% most heterozygous autosomal loci in different populations. (Yellow) European; (red) African; (blue) East Asian. The mean heterozygosities (dot) of the African subpopulations consistently exceed those of the non-African subpopulations. The whiskers extend to ±1 standard deviation. See Supplemental Table 3 for population abbreviations. (*B*) STRUCTURE clustering based on the 100 most polymorphic autosomal STR loci. Each subpopulation clusters tightly by geographic origin. Color labels as in *A*. (*C*) Average STR heterozygosity as a function of chromosome type. Bars denote the standard error.

To further assess the utility of our catalog, we tested its ability to replicate known population genetics trends. We specifically wondered about the quality of the most variable STR loci in the catalog. One hypothesis is that these loci are just extreme cases of genotyping errors; an alternative hypothesis is that these loci are truly polymorphic and can provide useful observations about the underlying populations. We first compared the heterozygosities of the 10% most variable autosomal loci across 10 different subpopulations from Africa, East Asia, and Europe. Consistent with the out-of-Africa bottleneck (Stoneking and Krause 2011), we found that the genetic diversity of the African subpopulations significantly exceeded those of Europe and East Asia (sign test; *P* < 10^−50^ for any African–non-African pair) (Fig. 3A; Supplemental Table 6). Second, we focused on the 100 most heterozygous autosomal loci in our catalog and inspected the ability of STRUCTURE (Pritchard et al. 2000) to cluster a subset of the samples into three main ancestries in an unsupervised manner. Our results show that all these samples clustered distinctly by geographical region (Fig. 3B). These analyses demonstrate that even the most variable loci in the catalog still convey valid genetic information that can be useful for population genetic analyses. Finally, we also analyzed the genetic variability of all STRs with MAF > 1% on the autosome, X chromosome, and Y chromosome (Fig. 3C). Autosomal STRs showed the highest variability, followed by STRs on the X and the Y chromosomes. This result is consistent with the differences in the effective population sizes of these three types of chromosomes, providing an additional sanity check.

In summary, the multiple lines of quality assessment suggest that our catalog can be used to infer patterns of human STR variations such as heterozygosity, allelic spectra, and population structure. The most notable shortcoming of the catalog is allelic dropouts stemming from the low sequencing coverage of the 1000 Genomes Project. However, the experiments above suggest that valuable summary statistics can be extracted from the call set despite this caveat.

### Patterns of STR variation

Despite a plethora of STR studies, there is no consensus in the literature regarding the effect of motif characteristics on STR variability. The classical study by Weber and Wong (1993) originally suggested that tetranucleotide STRs mutate more rapidly than those with dinucleotide motifs based on the analysis of de novo mutation in trios for 50 STRs. This finding was recently supported by a much larger trio-based study of nearly 2500 STRs (Sun et al. 2012). However, various other studies have suggested that dinucleotides have higher mutation rates (Chakraborty et al. 1997; Pemberton et al. 2009). These disagreements may largely stem from the fact that many of these studies considered very small panels of STRs that are subject to ascertainment biases.

**Figure 4.**
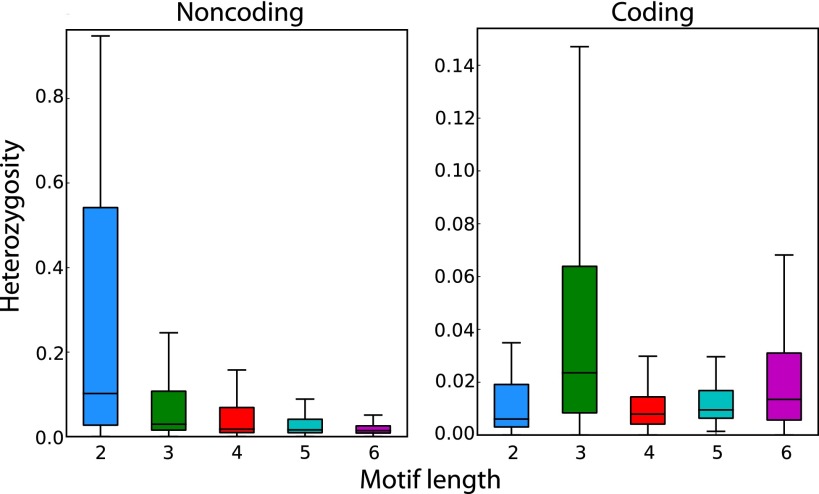
Motif length and coding capabilities as determinants of STR variability. STR heterozygosity monotonically decreases with motif length for noncoding loci and is generally reduced in noncoding (*left*) versus coding regions (*right*). The box extends from the lower to upper quartiles of the heterozygosity distribution, and the interior line indicates the median. The whiskers extend to the most extreme points within 1.5*IQR of the quartiles.

To address this open question, we analyzed the sequence determinants of STR variation in our catalog. We found that for noncoding STRs, variability monotonically decreased with motif length (Fig. 4). In contrast, loci with trimeric and hexameric motifs were the most polymorphic among coding STRs. These STR loci can vary without introducing frameshift mutations and therefore may be exposed to weaker purifying selection. In addition, coding STRs demonstrated significantly reduced heterozygosity compared to noncoding STRs for 2–5 bp motifs (Mann-Whitney *U*-test; *P* < 0.01) (Supplemental Table 7), whereas hexameric STRs showed no statistically significant difference in variability between these two classes. To ensure that the dependence between motif length and heterozygosity was not confounded by length or purity biases, we stratified STR heterozygosity for pure STRs based on major allele length and motif length. This analysis still showed an inverse correlation between motif length and STR variability after stratification based on the length of the most common allele (Supplemental Fig. 7). In addition, this analysis showed a monotonic increase in STR variability as a function of the major allele length. Similar trends also applied for STRs with various levels of impurities, albeit with a reduced magnitude of effect and slight deviations from monotonicity (Supplemental Fig. 8). This observation is concordant with previous studies (Ellegren 2000; Xu et al. 2000; Lai and Sun 2003; Whittaker et al. 2003).

Next, we explored the effect of nucleotide composition on STR variability, another issue for which the literature has not yet reached a consensus. Previous studies have suggested that AT repeats are the least variable motif for dinucleotide STRs (Bachtrog et al. 2000; Pemberton et al. 2009), whereas other studies claimed that AT repeats are the most variable motif (Kelkar et al. 2008; Sun et al. 2012). We repeated our analysis by stratifying the STRs based on motif sequence and major allele length (Supplemental Fig. 9). The resulting per-motif variability results were remarkably similar to those generated using a comparison of orthologous STRs in humans and chimpanzees (Kelkar et al. 2008). Our analysis shows that AT repeats are in general more variable that AC repeats after controlling for length of the most major allele. Similarly, for most motif lengths, STRs with an [A]_n_T motif tend to be more variable with long major allele lengths. However, we could not find a clear pattern across motif lengths, which is similar to the result of a previous analysis of a few dozen Y-STRs (Ballantyne et al. 2010).

### The prototypical STR

We also wondered about the prototypical pattern of variation of an STR locus in terms of the number of alleles and their distribution. We found that 30% of STRs have a common polymorphism with at least two alleles with frequencies >5%. Dinucleotide STRs have the highest rate, with 48% of these loci displaying a common polymorphism. Moreover, 30% of all dinucleotide STRs have more than three alleles with a frequency >5%. On the other hand, hexanucleotide STRs have the lowest common polymorphism rate, with only 13% of these loci displaying a common polymorphism (Supplemental Fig. 10A; Supplemental Table 8).

Next, we turned to finding the prototypal allelic spectra of STRs. For each STR, we normalized the reported alleles such that they reflected the distance in number of repeats from the locus’ most common allele. Then, we generated histograms that show the allelic spectra by aggregating all the alleles of STRs with the same motif length. This coarse-grained picture was similar across repeat lengths (Supplemental Fig. 10B). The allelic spectrum of an STR is unimodal and relatively symmetric. There is one, highly prevalent major allele, two less common alleles one repeat above and one repeat below the most common allele, and a range of rare alleles with monotonically decreasing frequency that reach more than ±5 repeats from the most common allele.

We also wondered about the population differentiation of autosomal STRs. We analyzed the Rst (Slatkin 1995) between African, Asian, and European populations for STRs with heterozygosity >5% (Supplemental Table 9). The average Rst was between 4.5% and 6% across the motif lengths and the median was ∼2%–3%. In coding regions, when compared to noncoding STRs, the average Rst was less than half for trimeric STRs but the same for hexameric STRs. Our results regarding population differentiation using STRs are reminiscent of a classical study that found similar levels of differentiation by analyzing close to 800 STR markers (Rosenberg et al. 2002).

### STRs in the reference genome and LoF analysis

**Figure 5.**
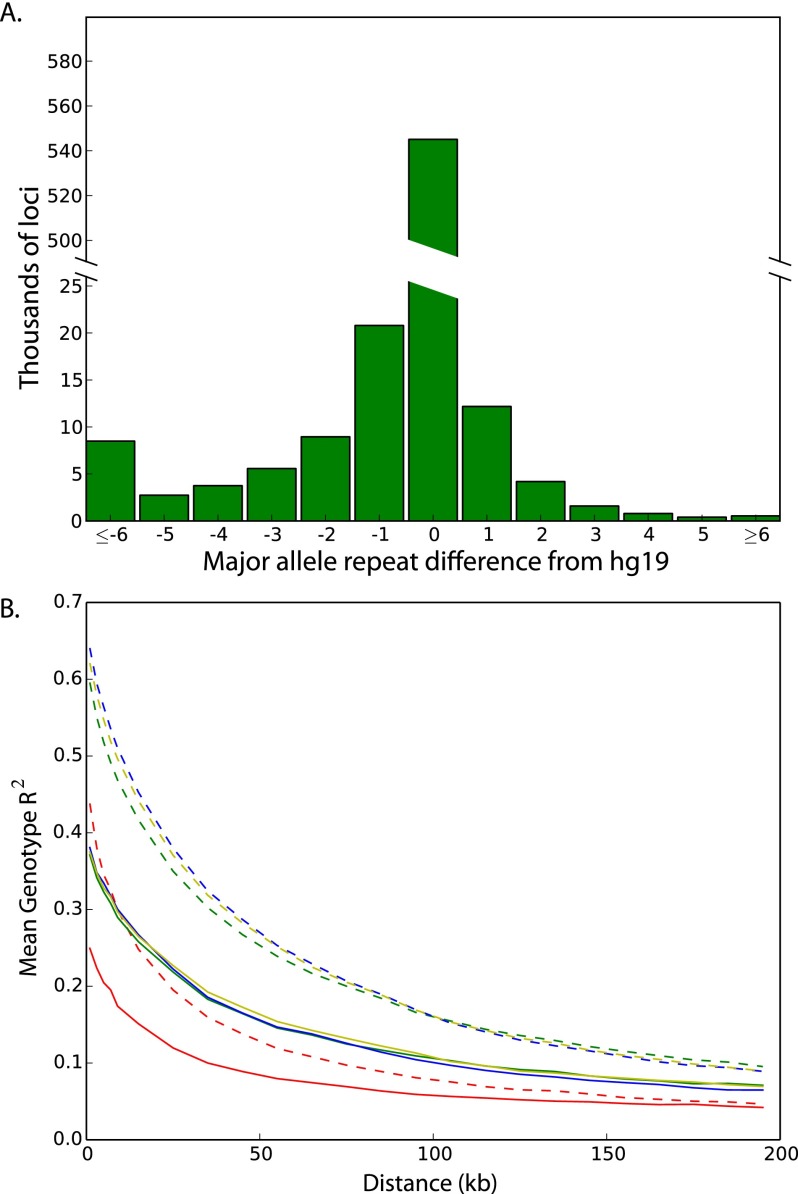
Population-scale analyses of STR variation. (*A*) Distribution of base-pair differences between each locus’ most common allele and the hg19 reference allele. (*B*) Patterns of linkage disequilibrium for SNPs and STRs on the X chromosome. SNP-SNP LD (dashed lines) generally exceeds SNP-STR LD (solid lines) across a range of distances for Africans (red), Admixed Americans (green), Europeans (yellow), and East Asians (blue).

We were interested in assessing how well the most common alleles are represented in the hg19 reference (Fig. 5A). We found that for more than 69,000 loci (10% of our reference set), the most common allele across the 1000 Genomes Project populations was at least one repeat away from the hg19 reference allele. Furthermore, the length of the most common allele only matched the length of the orthologous chimpanzee STR 50% of the time, reflecting the high mutability of these loci. In addition, 15,581 loci (2.25%) in the reference genome were 10 bp or more away from the most common allele in our data set.

For STRs in coding regions, the most common allele for 48 loci (1.1% of coding STRs) did not match the allele present in the hg19 reference (Supplemental Table 10). In 46 of 48 of these cases, these differences occurred for loci with trinucleotide or hexanucleotide repeats and conserved the reading frame. Moreover, for the two loci whose most common alleles were frame-shifted, these variations are unlikely to trigger the nonsense-mediated decay pathway. The deletion of one 4-bp unit in *DCHS2* occurs a few nucleotides before the annotated RefSeq stop codon. This variation slightly alters the location of the stop codon and affects only five amino acids in the C terminus of the protein. The 14-bp deletion in *ANKLE1* occurs in the last exon of the gene and introduces about 20 new amino acids into the tail of the protein.

We also sought to identify a confident set of STR loci with relatively common loss of function (LoF) alleles. To accomplish this goal, we considered only alleles supported by at least two reads and 30% of the total reads per called genotype. We further required that alleles be carried by 10 or more samples. Seven common LoF alleles across five genes passed this criterion: *DCHS2*, *FAM166B*, *GP6*, *SLC9A8*, and *TMEM254* (Supplemental Table 11). Of these five genes, only *GP6* has known implications for a Mendelian condition: a mild platelet-type bleeding disorder (Dumont et al. 2009; Hermans et al. 2009). However, the LoF mutation in this gene resides in the last exon and is unlikely to induce the nonsense-mediated decay pathway. In conclusion, the LoF analysis indicates that common STR polymorphisms rarely disrupt the reading frame.

### Linkage disequilibrium between STRs and SNPs

The linkage disequilibrium (LD) structure of STRs and SNPs is largely unknown. On top of recombination events, the SNP-STR LD structure also absorbs STR back mutations that could further shift these pairs of loci toward equilibrium. However, there is minimal empirical data in the literature about the pattern of this LD structure, most of which pertains to a few hundred autosomal Marshfield markers (Payseur et al. 2008). To get a chromosome-wide estimate, we inspected STR loci on the hemizygous X chromosomes in male samples. Similar to the Y chromosome data, these calls do not suffer from allelic dropouts and are already phased with SNP alleles, conferring a technically reliable data set for a chromosome-wide analysis.

We determined the LD in terms of the *R*^2^ between SNPs and STRs as a function of the distance between these markers. Only STRs and SNPs with common polymorphisms were used for the analysis. Hexameric STRs were not included due to the small sample size of 24 sites; for the other repeat motifs, we obtained hundreds to thousands of polymorphic markers. We stratified the STR-SNP LD based on the four major continental populations (Africa, Asia, Europe, and America) and contrasted them to the patterns for classical SNP-SNP LD (Fig. 5B). In all cases, the SNP-SNP LD consistently exceeded mean STR-SNP LD. In addition, the African population demonstrated markedly reduced levels of SNP-STR LD and SNP-SNP LD, consistent with its larger effective population size. In general, dinucleotide STRs showed the weakest LD with nearby SNPs, which likely stems from their higher mutation rates (Supplemental Fig. 11). To ensure that the reduction in STR-SNP LD did not stem from comparing *R*^2^ values for multiallelic and biallelic makers, we converted the STR alleles to binary markers, where the two states corresponded to the most common allele and all alternative alleles grouped together. The resulting levels of mean SNP-STR LD using these binary genotypes were nearly identical to those obtained using the multiallelic STR genotypes, indicating that this potential issue had little effect (Supplemental Fig. 12).

Overall, this analysis shows that the average SNP-STR LD is approximately half the SNP-SNP LD for variations with the same distance on the X chromosome. Since the effective population size of the X chromosome is smaller than that of the autosome, the STR-SNP LD should be even smaller on the autosome. These results suggest that association studies with tagging SNPs might be considerably underpowered to detect loci with causal STRs, specifically dinucleotide loci.

## Discussion

In the last few years, population-scale sequencing projects have made tremendous progress in documenting genetic variation across human populations. The 1000 Genomes Project has already reported ∼40 million SNPs, 1.4 million insertion and deletions, and more than 10,000 structural variants (The 1000 Genomes Project Consortium 2012). Similar catalogs, albeit to lesser degrees of completeness, have been produced for other types of variations, such as LINE-1 insertions (Ewing and Kazazian 2011) and *Alu* repeat variations (Hormozdiari et al. 2011). Here, we presented a population-scale analysis of STR variation, adding another layer of genetic variation to existing catalogs.

Our analysis significantly augments the level of knowledge of STR variation. Currently, dbSNP reports data for only 5500 STR loci. Our catalog provides data on close to 700,000 STR loci, which encompasses 97% of the STRs with motifs of 2–6 bp in the genome, and contains more than 300,000 STR loci with a MAF of >1%. One caveat of our catalog is the low reliability of individual genotypes due to allelic dropout. Nonetheless, we showed using multiple lines of analysis that reliable summary statistics such as frequency spectra and variation trends can be extracted from the catalog for most of the STRs. Another caveat of our catalog is that with the mixture of 76-bp and 100-bp sequencing reads, we could only unbiasedly ascertain the allelic spectra of ∼90% of the STRs, those with hg19 alleles of up to 45 bp. To indicate this caveat, our website alerts users about a potential bias in the allelic spectrum when inspecting STRs with reference allele length beyond this range. However, we expect the caveat will be alleviated in the near future with the public release of the Phase 3 data composed of a large number of Phase 1 samples resequenced with 100-bp Illumina reads. We expect that this data set will enable the generation of unbiased allelic spectra for longer STRs.

Despite these limitations, our data provide several biological insights about STR variation. Shorter repeat motif, longer major allele, higher purity of the repeat motif, and residing outside of a coding region are all associated with an increase in STR variability. Most of the STR loci display a unimodal distribution with one very common allele and a series of minor alleles with rapidly declining frequencies. This picture suggests that the stepwise mutation model largely describes the creation of new alleles in most of these loci. An open question is the exact mutation rate per generation for each locus in the genome. This question is theoretically addressable with a sufficiently large number of samples by analyzing the distribution of squared differences in the repeat size between two alleles of the same locus (Slatkin 1995). However, this question cannot be addressed by our call set due to the large number of allelic dropouts that might confound such an analysis and should be addressed with data sets obtained from deeply covered genomes.

The landscape of STR variation in the apparently healthy 1000 Genomes Project individuals suggests several rules of thumb for analyzing STR variations for medical sequencing. Previous work found that membrane proteins of several pathogens contain STR loci with nontriplet motifs whose variations can be beneficial to the organism (Gemayel et al. 2010). These STRs confer high evolvability and adaptability of these proteins by dynamically changing the reading frame. In contrast, our data suggest that for the vast majority of human proteins, frame-shift mutations in their STR regions are not favorable. Only a handful of STRs harbor common frame-shift polymorphisms, and half the LoF alleles create a very small change in the C-terminus of the protein. Based on these observations, we hypothesize that most of the non-triplet coding STRs are not well tolerated and are exposed to negative selection similar to regular indels in the same region. Therefore, it is advisable for medical sequencing projects to also analyze these loci and treat them as regular LoF alleles rather than filtering them. This rule of thumb is well-echoed in a recent study of medullary cystic kidney disease type 1 that implicated the genetic pathology in a frame-shift mutation caused by a length change of a homopolymer run (Kirby et al. 2013). For in-frame STR variations, our call set contains deep allelic spectra of most of these loci, providing reference distributions of apparently healthy alleles. These spectra can be used to identify atypical STR alleles and might serve as an indicator for pathogenicity.

Although STR alleles within our call set rarely induced frame-shifts, they may introduce premature stop codons by modulating the splicing machinery. Several prior studies have observed a direct dependence of splicing efficiency on STR repeat number for *CFTR* (Hefferon et al. 2004), *HTT* (Sathasivam et al. 2013), and *NOS3* (Hui et al. 2003). To facilitate the analysis of such cases, we created a dedicated table on the catalog website that specifies all 2237 STRs that reside within 20 base pairs of an exon–intron boundary.

Another issue raised by our findings is the potential contribution of STRs to complex traits. Using the prototypical allelic spectra, we estimate that the average variance of STR repeat dosage is 3, 0.7, 0.4, 0.25, and 0.1 for 2- to 6-mer STRs, respectively. Interestingly, the theoretical maximum variance for a biallelic SNP dosage is 0.5, six times smaller than the observed variance of dinucleotide STRs. From a theoretical statistical genetics perspective, this suggests that causal dinucleotide STR loci could explain a considerable fraction of phenotypic variance even with a relatively modest effect size. Therefore, if each STR allele in a locus slightly changes a quantitative trait in a gradual manner, the net effect on the phenotypic variance could be quite large due to the wide range of these alleles and their relatively high frequencies. Interestingly, we found that loci with dinucleotide motifs show relatively weak LD with SNPs, suggesting that GWAS studies with SNP arrays are prone to miss causal STR loci. Given the theoretical potential of STRs to contribute to phenotypic variance on one hand and their weaker LD to tagging SNPs on the other hand, one intriguing possibility is that STRs contribute to the missing heritability phenomenon of complex traits (Manolio et al. 2009; Press et al. 2014). Our hope is that this catalog can be a reference point to test this hypothesis in future studies.

## Methods

### Call set generation

The raw sequencing files for Phase 1 of the 1000 Genomes Project were analyzed.

The lobSTR calls were generated using computing resources hosted by Amazon Web Services, GitHub version 8a6aeb9 of the lobSTR genotyper, and Github version a85bb7f of the lobSTR allelotyper (https://github.com/mgymrek/lobstr-code). In particular, the lobSTR genotyper was run using the options fft-window-size=16, fft-window-step=4, and bwaq=15 and a default minimum flanking region of 8 bp on both sizes of the STR region. Reads that were aligned to multiple locations were excluded from the analysis. PCR duplicates were removed from the resulting BAM files for each experiment using SAMtools (Li et al. 2009). The individual BAMs were merged by population, and the lobSTR allelotyper was run using all population BAMs concurrently, the include-flank option, and version 2.0.3 of lobSTR’s Illumina PCR stutter model.

RepeatSeq (available at http://github.com/adaptivegenome/repeatseq) was run using default parameters on the read alignments produced by the 1000 Genomes Project. For both programs, we used the set of 700,000 STRs that was constructed using the second-order Markov framework (Supplemental Methods).

### Estimating the number of samples per locus and the number of loci per sample

The distributions of the call set parameters were smoothed using the gaussian_kde function in the *scipy.stats* python package. Covariance factors of 0.01 and 0.025 were used to smooth the samples per locus and loci per sample distributions, respectively.

### Saturation analysis

We determined the number of loci with calls for sample subsets containing 1, 5, 10, 25, 50, 100, 250, 500, 750, and 1000 individuals. In particular, we began by randomly selecting one individual. To create a subset of five individuals, we then added four more random individuals and so on. For each of these sample subsets, we determined the number of loci with one or more STR calls across all samples in the subset. We repeated this whole process 10 times and used the median number of called loci across each of the 10 repetitions to create the saturation profile for all loci.

We also determined whether loci had a MAF > 1% using all 1009 samples. We then used a procedure analogous to the one described above to select subsets of samples and determine whether or not each of these loci had a corresponding call in each subset. This procedure resulted in the saturation profile for loci with MAF > 1%.

### Mendelian inheritance

The three low-coverage trios contained within the data set consisted of the following sample sets: HG00656, HG00657, HG00702 (trio 1), NA19661, NA19660, NA19685 (trio 2), and NA19679, NA19678, NA19675 (trio 3). To assess the consistency with Mendelian inheritance for a given trio, only loci for which all three samples had calls were analyzed. The coverage assigned to each trio of calls corresponded to the minimum coverage across the three samples.

### Capillary electrophoresis comparison

Marshfield genotypes (Rosenberg et al. 2005) were downloaded from http://www.stanford.edu/group/rosenberglab/data/rosenbergEtAl2005/combined-1048.stru. Prior to comparing genotypes, offsets were calculated to match the lobSTR calls to the length of the Marshfield PCR products. For each locus, all observed offsets were considered and scored, and the optimally scoring offset across all samples was selected. In particular, for each sample, an offset was scored as a 1, 0.5, 0.25, or 0 if the lobSTR calls matched exactly, were homozygous and recovered one Marshfield allele, were heterozygous and recovered one Marshfield allele, or did not match at all, respectively. Only loci with at least 20 calls were considered in the comparison. Finally, the Pearson correlation coefficient was calculated using the sum of the allele length differences from hg19 for each locus in each sample.

Y-chromosome PowerPlex genotypes were downloaded from the 1000 Genomes Y chromosome working group FTP site. Offsets were once again calculated to match the length of the PCR products to the lobSTR calls. For each locus, the offset was calculated as the most common difference between the lobSTR and PowerPlex genotypes across samples. Only loci with at least five calls were considered in the comparison, and the *R*^2^ was calculated between the allele length differences from hg19 for each locus in each sample. In addition, the 15 heterozygous lobSTR calls were ignored.

Slopes and *R*^2^ values for STR dosage comparisons were calculated using the linregress function in the scipy.stats package. To mitigate the effects of outliers, we explored using regular linear regression, regression with a zero intercept, and L1 penalized regression. The resulting slopes were essentially invariant to the calculation method and so statistics were reported based on traditional linear regression.

### Heterozygosity calculations

For each analysis, heterozygosity was calculated using the aggregated frequency spectra according to the formula 
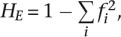
, where 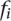 denotes the frequency of the *i*th allele at the locus.

### Summary statistic comparisons

The allelic spectra of the Marshfield panel were downloaded from http://research.marshfieldclinic.org/genetics/genotypingData_Statistics/markers/ and parsed using a custom Perl script (data and script available in Supplemental Material and at https://github.com/erlichya/str_catalog_supplemental_scripts). Samples from the CEU, GBR, TSI, and FIN subpopulations were analyzed, and only markers with more than 50 calls were included.

We utilized all of the lobSTR calls for the CEU, GBR, and FIN subpopulations to generate the lobSTR frequency spectra for each CODIS marker. Spectra were not available for three of the CODIS markers (D21S11, VWa, TPOX). D21S11 is too long to be spanned by Illumina reads; we had annotation difficulties for VWa and TPOX (assigning the correct STR in hg19 to the NIST STR). We then compared the available frequency spectra to those published for a Caucasian population in the United States (Budowle et al. 1999). Because of some annotation differences between the capillary data and our reference locations, we shifted the lobSTR spectra for the D8S1179 marker by +2 repeat units. Finally, repeat lengths for which the maximum frequency was <2% were not displayed.

### Comparison of population heterozygosity

To obtain accurate measures of heterozygosity, autosomal STR loci with less than 30 calls in any of the 10 subpopulations considered were ignored. Of the remaining loci, the 10% most heterozygous (24,637 loci) were selected, and their means and standard deviations were calculated. To determine whether a pair of populations had systematically different heterozygosity at these loci, we paired the heterozygosities for each locus and counted the number of pairs in which population A had a larger heterozygosity than population B. Ignoring the relatively small number of loci in which heterozygosities were identical, the *P*-value for this over/underrepresentation was then calculated using the cdf function in the scipy.stats.binom python package.

### Deviation of lobSTR calls from the hg19 reference

For each locus with one or more genotyped samples, we calculated the mean deviation of all samples’ genotypes from the hg19 reference allele. We then pooled these per-locus deviations by reference allele length using 5-bp intervals. The median within each length bin resulted in the corresponding plot of deviation versus reference allele length.

### Sample clustering

STRUCTURE version 2.3.4 was utilized to perform the MCMC-based clustering (Falush et al. 2003). The program was run using MAXPOPS=3, BURNIN=500000, NUMREPS=1000000, no prior population information, unphased genotypes, the admixture model, and no linkage disequilibrium. All 321 samples from the JPT, CHB, YRI, and CEU subpopulations present in the data were clustered based on the 100 most heterozygous autosomal STRs with at least 750 called samples. Samples for which at least 75% of the selected makers were missing calls were not included in the resulting visualization. The final triangle plot therefore contained data for 71, 80, 81, and 82 samples from the CEU, CHB, JPT, and YRI populations, respectively.

### STR variability trends

Analysis was restricted to STRs with at least 100 called samples. STRs that overlapped an annotated RefSeq translated region were regarded as coding, and these annotations were downloaded from the UCSC table browser on February 11, 2014. The mannwhitneyu function in the scipy.stats python package was used to test for significant differences between coding and noncoding STR heterozygosity. For analyses related to allele length or purity, STRs were further restricted to those whose most common allele matched the hg19 reference to enable calculation of the locus’ purity. In particular, the purity of each of these STRs was calculated as the fraction of possible positions within the STR region where the subsequent bases corresponded to a cyclic permutation of the STR’s motif. The *pearsonr* function in the *scipy.stats* python package was used to calculate the Pearson correlation coefficients and their associated *P*-values, where each STR’s length and heterozygosity represented an individual point. Finally, to generate the plots of heterozygosity versus length, the heterozygosity for each length was calculated as the mean variability of loci within 2 bp.

### Extraction of orthologous chimpanzee STR lengths

Tandem Repeats Finder was run on the panTro4 assembly of the chimp genome using the default parameters and a minimum score threshold of 5. To resolve overlapping repeats, we discarded repeats with period greater than six and scanned from low to high coordinates and selected the highest scoring repeat for each overlap conflict. The chimp coordinates were mapped to hg19 coordinates using liftOver and a minimum mapping fraction of 50%. We then intersected these coordinates with those of our reference panel and retained those loci within our panel that had a single intersecting chimp repeat whose motif matched. This resulted in orthologous chimp repeats for ∼83% of our reference set of STRs.

### Rst levels

The Rst was calculated according to Slatkin (1995) using a custom Python script (code available in Supplemental Material and at https://github.com/erlichya/str_catalog_supplemental_scripts). The African, European, and Asian populations were comprised of the same subpopulations used throughout this study, except that the ASW population was omitted due to potential admixture. Only loci with heterozygosity >5% and at least 100 genotyped samples were considered.

### Assessing linkage disequilibrium

In order to avoid phasing SNPs and STRs, we only analyzed X chromosome genotypes in male samples. SNP calls for the corresponding samples were obtained from the 1000 Genomes Project Phase 1 November 23, 2010, release and any pseudoautosomal loci were ignored. Analysis of STR-SNP LD was restricted to STR loci with both a heterozygosity of at least 9.5% and at least 20 genotypes for each super population (African, East Asian, European, and Admixed American). For each STR that met this requirement, we identified all SNPs within 200 kb of the STR start coordinate. After filtering out SNPs with a MAF < 5% in any of the four super populations, we calculated the level of LD for the remaining STR-SNP pairs. In particular, the *R*^2^ was calculated between the SNP genotype indicator variable and the base pair difference of the STR from the reference. We also recalculated the STR-SNP LD after converting the STR alleles to binary variables, where the most common allele and all alternative alleles were mapped to 0 and 1, respectively. This binary mapping was applied to each super population individually.

For SNP-SNP LD calculations, a seed SNP was identified for each STR meeting the aforementioned requirements. In particular, the SNP closest to the STR’s start coordinate with MAF > 5% for each super population was selected. If no such SNP existed within 1 kb, no SNP was selected and the STR was omitted from the STR-SNP LD analysis. Otherwise, we identified all SNPs within 200 kb of the seed SNP and once again removed SNPs with a MAF < 5% in any of the super populations. The LD between the seed SNP and each of these remaining SNPs was then assessed as the *R*^2^ between the two SNP genotype indicator variables.
